# Electroporation of mice zygotes with dual guide RNA/Cas9 complexes for simple and efficient cloning-free genome editing

**DOI:** 10.1038/s41598-017-18826-5

**Published:** 2018-01-11

**Authors:** Marie Teixeira, Bénédicte F. Py, Christophe Bosc, Daphné Laubreton, Marie-Jo Moutin, Jacqueline Marvel, Frédéric Flamant, Suzy Markossian

**Affiliations:** 1 SFR BioSciences, Plateau de Biologie Expérimentale de la Souris (AniRA-PBES), Ecole Normale Supérieure de Lyon, Université Lyon1, CNRS UMS3444, INSERM US8, 69007 Lyon, France; 2 CIRI, INSERM U1111, Université Claude Bernard Lyon 1, CNRS UMR 5308, École Normale Supérieure de Lyon, Université de Lyon, 69007 Lyon, France; 3 Université Grenoble Alpes, Grenoble Institut des Neurosciences, GIN, F-38000 Grenoble, France; 4 INSERM, U1216, F-38000 Grenoble, France; 5 Institut de Génomique Fonctionnelle de Lyon, INRA USC 1370, Université de Lyon, Université Lyon 1, CNRS UMR 5242, Ecole Normale Supérieure de Lyon, 46, allée d’Italie, 69007 Lyon, France

## Abstract

In this report, we present an improved protocol for CRISPR/Cas9 genome editing in mice. The procedure consists in the electroporation of intact mouse zygotes with ribonucleoprotein complexes prepared *in vitro* from recombinant Cas9 nuclease and synthetic dual guide RNA. This simple cloning-free method proves to be extremely efficient for the generation of indels and small deletions by non-homologous end joining, and for the generation of specific point mutations by homology-directed repair. The procedure, which avoids DNA construction, *in vitro* transcription and oocyte microinjection, greatly simplifies genome editing in mice.

## Introduction

Since its adaptation to mammalian cells^1,2^, CRISPR/Cas9 genome editing has become a key element of the genetic toolbox in mice^3,4^. The method relies on *Streptococcus pyogenes* Cas9 (SpCas9), a RNA-guided DNA endonuclease. Annealing between genomic DNA and the 5′-end of the guide RNA defines the position of the cleavage site by SpCas9, provided that the homologous genomic DNA sequence is immediately followed by a so-called protospacer adjacent motif (PAM: 5′-NGG-3′). SpCas9 cleaves both DNA strands 3 nucleotides (nt) upstream to the PAM. Therefore, the sequence of the first 20 nt of the guide RNA defines the genomic location of the double strand break^5^. Cells can repair double strand breaks in two ways: (1) non-homologous end-joining (NHEJ), an error prone mechanism which often generates small insertions and deletions (indels) at the cleavage site, (2) homology-directed repair (HDR) which uses an intact DNA molecule as repair template. The repair template can be provided as a single-stranded oligodeoxynucleotide (ssODN) to generate point mutations or small insertions. In that case, HDR introduces predefined mutations, and therefore can be used to precisely modify coding sequences.

**Figure 1:**
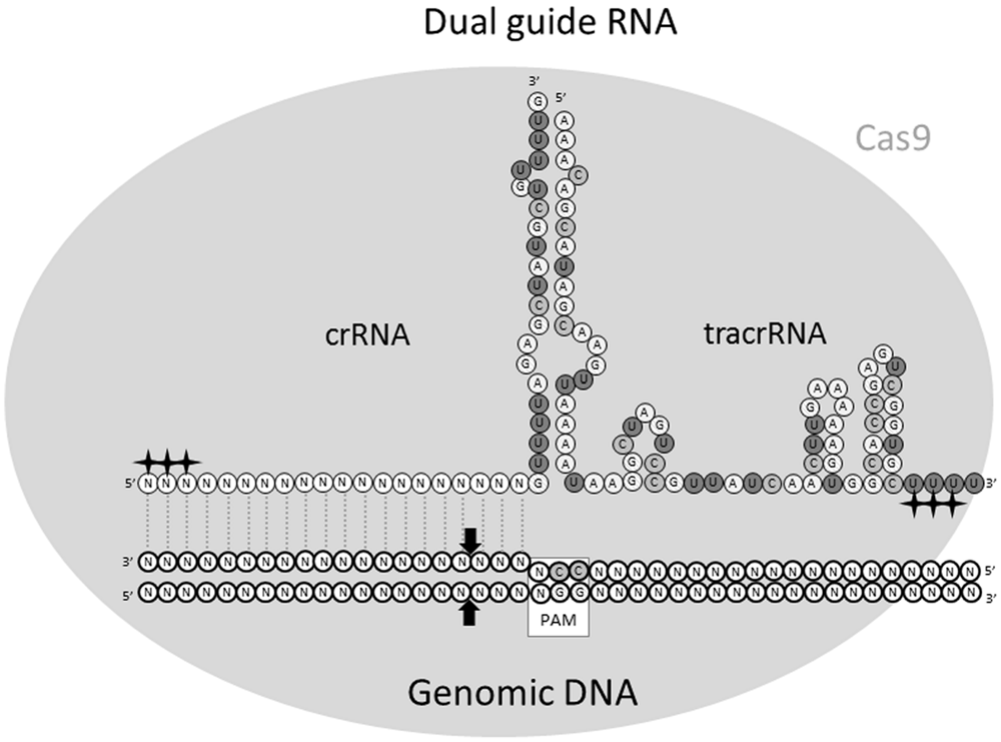
General structure of the dual guide RNA. Two synthetic RNA molecules are annealed. The short variable 42mer called crRNA has its first 20 nt designed to match a genomic sequence located immediately upstream to a PAM consensus motif (5′NGG) and the sequence is identical to the bottom genomic DNA strand on this figure. The second RNA, called tracrRNA, is a constant 72mer with partial homology to the crRNA, which secondary structure permits the tight association to the Cas9 nuclease (grey). Black crosses indicate the modified nucleotides, to which 2′-O-methyl and 3′ phosphorothioate are added to protect from nuclease degradation. Arrows indicate the cutting sites of the Cas9 nuclease.

The methodology for CRISPR/Cas9 mediated genome editing is improving rapidly to increase in simplicity, efficiency and versatility, as exemplified by the recent development of cloning-free procedures. They combine the use of recombinant Cas9 protein with chemically synthetized guide RNA^6^, usually in the form of a dual guide RNA^7^ obtained by annealing two RNA molecules: a long component (72-mer) with a constant nucleotide sequence called tracrRNA and a short variable component (42-mer) called crRNA. The 5′ part (20 nt) of the crRNA is homologous to the targeted genomic DNA sequence, while its 3′ part is complementary to the tracrRNA (Fig. 1). Using a dual guide RNA, rather than a single guide RNA (sgRNA), circumvents the difficult chemical synthesis of long RNA molecules and is cost-effective since the same batch of tracrRNA can be aliquoted and used for several projects. Chemical modifications introduced at the extremities of the RNAs protect from exonucleases, and significantly improve efficiency^6,8^. When mixed *in vitro*, SpCas9 and the dual guide RNA assemble into ribonucleoprotein complexes (RNPs) which can be introduced into mouse zygotes by microinjection^7^ or in cultured cells by electroporation^9,10^.

Electroporation can also be efficiently used to introduce RNP complexes made of Cas9 and sgRNA into mouse zygotes^11–13^. In the present manuscript, we show the clear advantage of combining electroporation, with the cloning-free procedure using dual guide RNA/SpCas9 RNP complexes to perform genome editing in mice. We found that these RNPs electroporation can generate mutations by NHEJ with 100% efficiency. The coelectroporation of two different RNPs can be used to generate deletions, or to simultaneously generate small indels at two different *loci*. If a ssODN is added to the RNPs preparation in order to provide template for HDR, the procedure also leads to the generation of specific point mutations. Overall, this new protocol is cost-effective and makes CRISPR/Cas9 in mice amenable for small facilities and laboratories without previous expertise in mouse transgenesis.

## Results

The four following experiments of genome editing were recently performed in our facility by combining the cloning-free procedure and zygote electroporation as depicted in Table 1. Technical details are presented in the method section, while all RNA and DNA sequences are detailed in Supplementary Table S1.

**Table 1:** General cloning-free procedure for zygote electroporation.

**Dual guide RNA synthesis**. Design and order crRNA: 5′[N*N*N*]NNNNNNNNNNNNNNNNN GUUUUAGAGCUAUGCUGUUUUG3′ (Ns are copied from a genomic sequence upstream to a PAM consensus: 5′NGG). Mix with tracrRNA 5′AAACAGCAUAGCAAGUUAAAAUAAGGCUAGUCCGUUAUC AACUUGAAAAAGUGGCACCGAGUCGGUGC[U*U*U*]U3′) and store 4 µL aliquots (1 µg/µL) at −80 °C (N.B: [] are for 2′-O-methyl and * are for 3′phosphorothioate modifications).
**Embryos harvest**. Release zygotes from oviducts in 200 μL droplets of Hyaluronidase /M2 medium. Incubate for 1 min to dissociate zygotes from follicular cells. Pass isolated zygotes through three washes in M2 medium using a mouth-pipette. Keep zygotes in M16 medium in incubator (5% CO_2_, 37 °C) until electroporation.
**RNPs assembly**. Thaw 4 µL of dual guide RNA preparation (1 µg/µL). Denature (2 min; 80 °C) and anneal (10 min; 37 °C). Add 2 µL of recombinant SpCas9 protein (5 µg/µL) at room temperature. Mix by pipetting and leave 10 min at room temperature. Add 1 µL of ssODN (4 µg/µL) if required. Adjust to 20 µL with OptiMEM medium.
**Electroporation**. Align 15–30 zygotes in the glass chamber, between 1 mm gap platinum plate electrodes filled with 5 μL of RNPs solution. Adjust volume to keep impedance between 120 and 180 kΩ. Apply poring and transfer pulses as described in Methods.
**Embryos reimplantation**. Transfer electroporated embryos in Petri dish with central well containing M16 medium and keep overnight in CO_2_ incubator. Implant 2-cell embryos into the oviduct of B6xCBA/F1 pseudopregnant females.

### Generation of small indels by NHEJ repair after electroporation of RNPs

**Figure 2:**
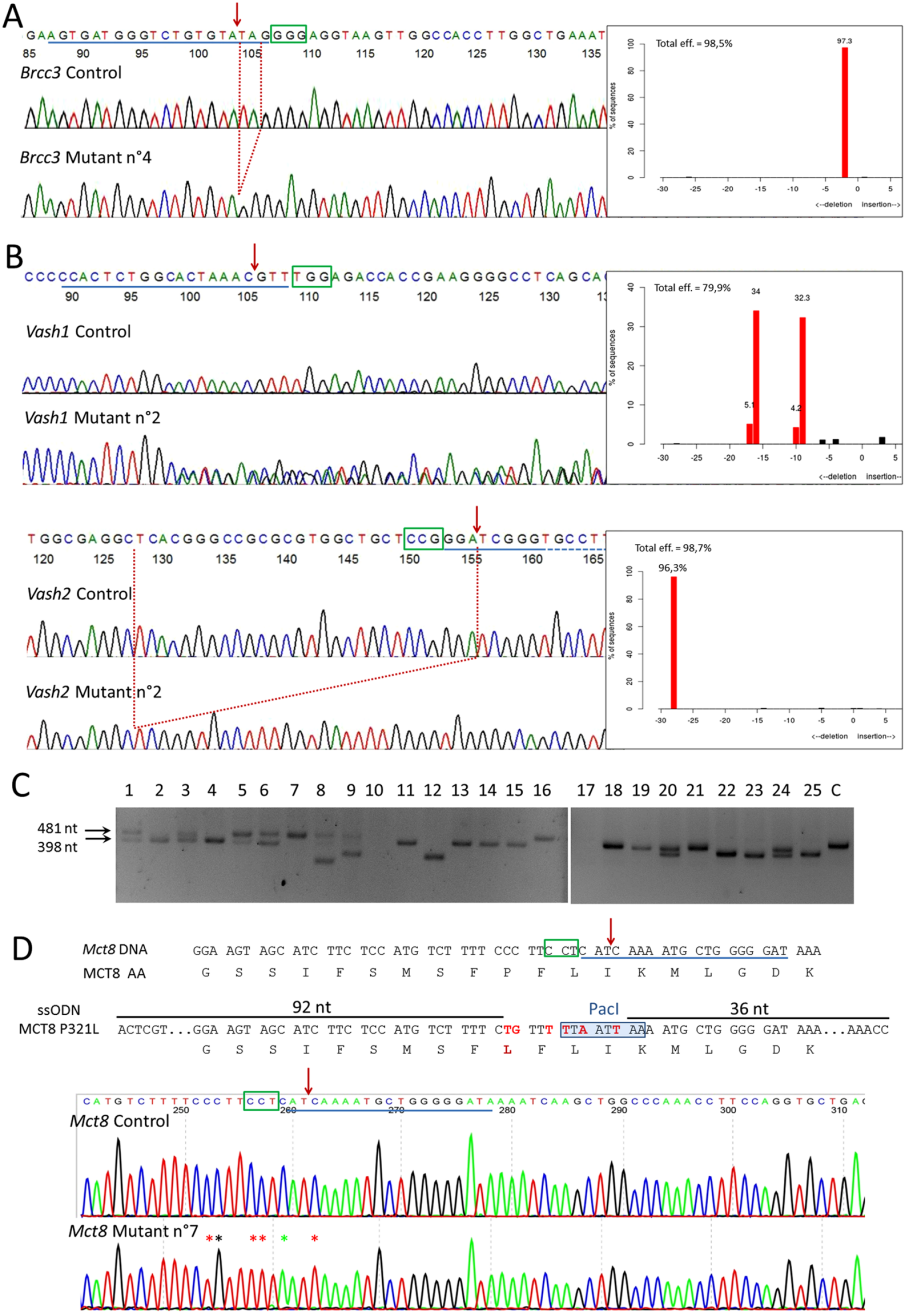
Mice genotyping (**A**) Use of a dual guide RNA to generate short indels on *Brcc3* gene. DNA Sanger sequencing of control and mutated F0 n°4 mice are shown. Top chromatogram represents a part of wild-type *Brcc3* gene: guide sequence is underlined in blue, green rectangle indicates PAM, red arrow indicates Cas9 cutting site. Bottom chromatogram represents mutant mouse n°4, in which NHEJ generated a 2 nt deletion (dotted red lines) without apparent mosaicism, as correlated by TIDE software analysis represented on the right side of chromatograms. (**B**) Simultaneous use of two dual guide RNAs to generate short indels on two vasohibin genes located on different chromosomes. DNA Sanger sequencing of control and mutated F0 mice n°2 are shown for *Vash1* (top chromatograms) and *Vash2* (bottom). The TIDE software is used to analyze the spectrum and frequency of indels, as represented on the right side of chromatograms. On control sequences, guide sequences are underlined in blue and green rectangles indicate PAM. Red arrows indicate Cas9 cutting site. On *Vash1* mutant lane, mosaicism and heterozygosity generate a scrambled sequence. *Vash2* mutant chromatogram represents a case without apparent mosaicism, in which NHEJ generated a 28 nt deletion (dotted red lines) on both *Vash2* alleles. (**C**) Simultaneous use of two dual guide RNAs to generate a 83 bp deletion in *Ctse* gene. Agarose gel analysis (cropped picture) of PCR performed on F0 mice genomic DNA, showing the frequent presence of a shorter PCR product of the expected size: the intended deletion of 83 bp leaves 398 bp instead of the 481 bp for the wild-type allele. Although most mice seem to harbor the expected deletion, additional small indels were identified by Sanger sequencing for some of them. Samples 8, 9 and 12 have larger deletion. Samples 10 and 17 repeatedly failed to amplify, suggesting large deletion, as DNA integrity was verified with primers targeting an independent locus (Supplementary Fig. S3). C represents control mouse with only the 481 bp wild-type allele. Full-length gels are presented in Supplementary Fig. S3. (**D**) Generation of specific point mutations using ssODN as template for HDR. A fraction of the *Mct8* genomic sequence and the corresponding reading frame are shown. The sequence underlined in blue is complementary to the 5′ end of the crRNA. The green rectangle indicates the complement of the PAM sequence. Red arrow indicates Cas9 cutting site. The ssODN has perfect homology for the *Mct8* gene and is identical to the “non PAM” strand on 92 nt at the 5′ end and 36 nt at the 3′ end. Mismatches are with red letters. The first two mismatches change the proline codon into a leucine codon. The following ones eliminate the PAM sequence, preventing Cas9 cleavage after HDR, and create a Pac I restriction site facilitating mouse genotyping. Top chromatogram represents control mouse sequence and the bottom one represents mutated F0 male n°7. Asterisks indicate mutated nucleotides.

The aim of the experiment was to generate frameshift mutations in order to inactivate the function of *Brcc3* gene (encoding subunit 3 of the BRCA1-BRCA2-containing complex), a gene located on the X chromosome. RNPs directed against *Brcc3* gene were electroporated into zygotes. DNA samples were prepared from 27 pups born after embryo reimplantation. The DNA sequence surrounding the expected double strand break was amplified by PCR and analyzed by agarose gel electrophoresis to identify major changes in fragment size. Large deletions were detected in two DNA samples (close to 200 bp, Supplementary Fig. S1). Four DNA samples repeatedly failed to amplify, suggesting the presence of larger deletions, as DNA integrity was verified with primers targeting an independent locus. This hypothesis was confirmed for three samples by an additional PCR analysis targeting a larger sequence (Supplementary Fig. S1). Sanger sequencing and TIDE analysis were performed for the 21 remaining samples in which deletions were not obviously visible. We identified small indels in the genome of all the corresponding pups (Fig. 2A, Table 2). This analysis failed to reveal the persistence of the wild-type allele in the somatic tissues of the F0 pups. In 20 out of these 21 mice, up to two alleles were detected (Table 3), reflecting mosaicism, at least in male pups. This is caused by NHEJ-generated mutations occurring after the first mitotic cell division of the embryo. Males n°3 and 8 were mated with wild-type C57BL/6 mice. As anticipated, genotyping of the female offspring revealed the germline transmission of one mutant allele to each pup (n = 6 and n = 4). F0 mice transmitted only one of the two mutant alleles detected in somatic tissues. Surprisingly, the allele transmitted by mouse n°8 was the least frequent in somatic tissue (8.9%), according to TIDE analysis. Therefore, information from genotyping of F0 toe clips cannot always be extrapolated to predict the germline genetic content.

**Table 2:** Embryo viability and editing efficiencies.

Gene	Type of mutation	Electrop. Zygotes	Transfered embryos (%)	Newborns (% of transfered embryos)	Analyzed F0	Mice with mutations (%)	Detected allele (number of mice)				
							WT	Indel	83 bp deletion	Point mutations (HDR)	Others^c^
*Brcc3*	Small indels	101	94 (93%)	27 (29%)	27	100%	0	21 (78%)	n/a	n/a	6
*Vash1* & *Vash2*	Small indels	45	42 (93%)	12 (29%)	11	100%	4	11^a^ (100%)	n/a	n/a	1
*Ctse*	Deletion	140	124 (89%)	25 (20%)	25	100%	nd	5^b^	15 (60%)	n/a	5
*Mct8*	Point mutations	91	88 (97%)	16 (18%)	16	100%	0	3	n/a	11 (69%)	2

n/a: not applicable; nd: not determined; ^a^mice with indel on each targeted gene; ^b^mice with indel at only one of the targeted sites.

^c^larger deletion for *Brcc3* and *Ctse*; supplemental larger insertion for *Vash1*; partial ssODN integration for *Mct8*.

**Table 3:** TIDE analysis of mosaicism after mutation of a X-linked gene.

**Mice**	**Sex**	**Insertion (size nt and %)**	**Deletion (size nt and %)**
***Brcc3***			
1	F	1 (4.9%); 2 (71.3%)	7 (9.9%)
3	M		2 (81.4%); 4 (3.9%)
4	M		2 (96.9%)
6	F	1 (39.6%)	16 (53.8%)
7	F	1 (45.8%)	2 (47.7%)
8	M		1 (8.9%); 13 (88.4%)
9	M		12 (96.4%)
11	M		12 (95.8%)
10	M	1 (82.1%)	1 (3.1)
12	F		1 (44.1%); 2 (50.3%)
13	M	1 (26.3%); 2 (53.3%)	
14	M		2 (96%)
16	M	1 (73.3%)	8 (23.2%)
18	M		12 (49.4%); 21 (44.8%)
19	M		2 (84.1%); 4 (3.3%)
20	M		13 (80.5%); 16 (4.3%)
22	F	1 (42.1%)	2 (44.5%)
23	M		8 (91.5%); 23 (5.8%)
24	M		12 (83.3%); 14 (3%)
25	M	2 (83.1%)	1 (4%)
26	M	1 (43.2%)	2 (40.1%)

### Generation of small indels on two loci by NHEJ repair after electroporation of RNPs

The aim of the experiment was to generate frameshift mutations in order to inactivate the function of both *Vash1* and *Vash2* genes (encoding vasohibin 1 and vasohibin 2) located on two different chromosomes. RNPs directed against each gene were electroporated together. Sequences surrounding the expected double strand breaks were amplified by PCR from 11 mice. Agarose gel electrophoresis did not identify major changes in fragment size, except for one mouse, where a large insertion was found (Supplementary Fig. S2). Sanger sequencing identified indels in both *Vash1* and *Vash2* genes for all analyzed newborns (Fig. 2B and Table 2). Deletion of up to 35 nt and 42 nt were respectively found for *Vash1* and *Vash2* (Table 4). By TIDE analysis, we identified the wild-type allele in only 4 pups. We identified several mutations in each newborn (2 to 5 different alleles identified for *Vash1* gene and 1 to 3 alleles for *Vash2* gene), revealing mosaicism. Two F0 mice were mated with wild-type C57BL/6 mice for germline transmission. Only 2 F1 pups from breeder n°7 were genotyped, and both inherited *Vash1* and *Vash2* mutations. Mice n°6 produced F1 offspring in which all pups (n = 15) inherited one mutant allele for *Vash1* and one mutant allele for *Vash2* genes, confirming the complete absence of wild-type alleles in the F0 mice.

**Table 4:** TIDE analysis of mosaicism after simultaneous mutations of two genes.

**Mice**	***Vash1***			***Vash2***		
	**Insertion** (size nt and %)	**Deletion** (size nt and %)	**remaining wild-type allele**	**Insertion** (size nt and %)	**Deletion** (size nt and %)	**remaining wild-type allele**
1		18 (38%); 29 (11%); 35 (42%)	0%		42 (40%)	18%
2		9*(32%); 10 (4%); 16 (34%); 17 (5%)	0%		28*(96%)	0%
3		1* (11%); 2 (8%); 21 (28%); 22 (7%)	0%		11*(35%); 27 (59%)	0%
4		15 (47%)	43%		6*(30%); 40(57%)	0%
5		17 (51%); 18(12%)	0%		1 (50%)	47%
6		14 (9%); 24 (7%); 25 (35%)	0%	2 (47%)	6*(50%);	0%
7	1*(62%)	14(33%)	0%		1*(21%); 2 (50%); 4 (25%)	0%
8		15(20%); 27(33%); 28(8%)	0%		37*(10%); 39 (33%)	0%
9		16*(34%); 27(57%)	0%	1 (43%)	7* (51%)	0%
10		2(10%); 18(5%)	53%	1 (37%)	2 (14%)	46%
11		2*(20%); 10 (9%); 12 (24%); 13 (5%); 16 (4%)	0%	1 (10%)	7 (38%)	0%

*spacing not constant.

### Generation of a deletion by coelectroporation of two RNPs

Our goal was to generate a 83 bp deletion in the *Ctse* gene, encoding Cathepsin E. This was achieved by co-introducing two RNPs targeting neighboring sequences. Analysis of PCR products by agarose gel electrophoresis indicated the presence of a deletion with the approximate expected size in 15 out of 25 mice (Fig. 2C). Larger deletions, up to 230 bp, were identified for 3 mice (n°8, 9 and 12). PCR amplification failed repeatedly for 2 mice suggesting the presence of large deletions, as DNA integrity was verified with primers targeting an independent locus (Supplementary Fig. S3). Sanger sequencing of PCR products confirmed gel analysis results regarding deletions, and identified 5 mice with small indels on one or both targeted sites (Table 2). Only the 83 bp deletion, and no other allele, was identified in 9 out of 23 mice, suggesting the deletion of both alleles in all cells (Supplementary Fig. S3). Two of these 9 F0 mice were mated with wild-type mice for germline transmission of the deleted allele. The result of the first litter, in which 6 out of 11 pups inherited the mutation, was finally indicative of germline heterozygosity or mosaicism in the F0 mice. In the second litter, all individuals (n = 8) were heterozygous for the deletion, suggesting germline homozygosity in the F0 mice.

### Introduction of in frame missense mutation by HDR from an exogenous single-stranded DNA template

We used HDR to introduce an amino-acid substitution (P321L) in the MCT8 transporter, encoded by *Mct8* located on the X chromosome. The dual guide RNA was designed to cut the DNA sequence next to codon 321. Several mismatches were introduced in the ssODN template: (1) the first two mutated one codon introducing P321L, (2) a silent mutation eliminated the PAM sequence from the genome preventing further Cas9 cleavage after HDR, (3) silent mutations of 3 nucleotides created a PacI restriction site to facilitate genotyping (Fig. 2D). Allele-specific PCR and PCR/Sanger sequencing identified all the designed mutations in 69% of the pups (Fig. 2D, Table 2 and Supplementary Fig. S4). We also found 2 unusual cases in which a portion of the ssODN sequence was inserted at the Cas9 cleavage site by NHEJ. Of note, only the expected mutant allele, and no other allele, was detected in 5 males and 3 females by TIDE analysis, suggesting that there was no mosaicism in these F0 mice and that they were hemizygous males and homozygous females. Absence of mosaicism in the germline was confirmed for 3 of these F0 males. Indeed, all F1 female offspring (n = 5; n = 6; n = 8) from each of these mutated F0 males were heterozygous for the mutation. On the contrary, offspring analysis from one F0 female was rather suggestive of mosaicism or heterozygosity in this female. Indeed, only 5 out of 12 pups inherited the mutation. We also successfully verified the absence of a non-intended mutation on a predicted intronic off-target site on the X chromosome (Supplementary Fig. S4 and Table S2).

Altogether these data show the outstanding performance of this new procedure combining zygote electroporation with the use of dual guide/SpCas9 RNP complexes.

## Discussion

A number of possible combinations have been used for CRISPR/Cas9 genome editing in mice. Cas9 can be encoded by a microinjected plasmid^14^, a microinjected mRNA^15^, or directly provided as a recombinant protein^7,16,17^. Single guide or dual guide^10^ RNA can be used, transcribed either *in vivo* from a microinjected plasmid or *in vitro* with a phage polymerase. Guide RNA can also be produced by chemical synthesis^10^. Finally, microinjection can be substituted by electroporation^11–13,18–20^, which can also be performed *in situ* in oviduct^21^. Here, the presented examples illustrate the efficiency of mouse zygotes electroporation with RNPs composed of recombinant Cas9 and synthetic dual guide RNA with chemical modifications. This strategy enables to achieve genome editing in mice with unprecedented simplicity. Although we did not perform side by side comparisons, it seems that this protocol outperforms the other methods that we have tested (Supplementary Fig. S5). Comparison of the mutation rates that we obtained with published results also supports the high performance of this new method^12,13,22^. In particular, the possibility to achieve HDR in the majority of the treated embryos is impressive. Although the cloning-free procedure does not eliminate mosaicism, which is a limitation common to all current methods, we observed the complete elimination of the wild-type allele in most experiments. In some cases, only one mutant allele was detected, suggesting that all cells were homozygous for the desired mutation. However, later analysis showed that the apparent homozygosity of somatic tissue biopsies does not necessarily imply homozygosity of the germline. If mosaicism has to be reduced, one possible improvement would be to perform electroporation at earlier developmental stage^12^. We also found that two different genes can be mutated independently in a single experiment, a non-negligible advantage on both ethical and financial points of view. Indeed, it reduces the number of animals needed and can significantly improve the throughput of genome editing facilities. Nevertheless, this strategy has limitation as multiplexing might increase the chances of generating chromosomal translocation^23–25^.

The other limitation of genome editing is the likely occurrence of off-target mutations, which we did not specifically addressed here. Although the occurrence of off-target mutations seems to be only partially predictable^26^, these can be kept infrequent by proper guide RNA design. Here we used the CRISPOR algorithm to optimize the design, without favoring efficiency over specificity. It should be stressed however that off-target mutations do not raise major problems for later analysis, provided that mouse phenotyping is performed only after generation F2. In this case, mutations localized far enough from on-targets will segregate during mouse mating. The current procedure can still be improved to limit the frequency of off-target mutations, since modified Cas9 nuclease with increased specificity^27–29^ become commercially available as recombinant proteins.

One attractive aspect of the cloning-free procedure is that it does not require any specific expertise in recombinant DNA manipulation and notably in *in vitro* transcription. Producing high quality guide RNA with bacteriophage RNA polymerases often appears as a limiting factor. RNA chemical synthesis also facilitates the introduction of chemical modifications which increase the genome editing efficiency by protecting dual guide RNA from degradation.

Dual guide RNPs electroporation of fertilized oocytes, with intact *zona pellucida*, is feasible with limited equipment, technical skills and financial resources. The technology is thus amenable to facilities which are familiar with embryo transfer, but not with molecular cloning or oocyte microinjection. Moreover, the remarkable efficiency of this method allows reducing the number of mice required to generate genetically modified models. It should also be easy to transpose to other mammalian species whenever embryo electroporation is possible^18,30^.

## Methods

The research projects were approved by a local ethics committee (CECCAPP, registered as CEEA015 by the French ministry of research) and subsequently authorized by the French ministry of research. All procedures were in accordance with the European Community Council Directives of September 22, 2010 (2010/63/EU) regarding the protection of animals used for scientific purposes.

### Embryo collection

Embryos from several genetic backgrounds were used: C57BL/6 J for *Brcc3* gene edition, B6D1F1xFVB or FVBxFVB for *Vash1* and *Vash2* genes, B6.SJL-PtprcaPepcb/BoyCrl for *Ctse* gene and B6D2F1xFVB for *Mct8* point mutations. Four or five weeks old female mice (Charles River, France) were superovulated by intraperitoneal (i.p.) administration of 5 IU of Pregnant Mare Serum Gonadotropin (PMSG, Alcyon, France), followed by an additional i.p. injection of 5 IU Human Chorion Gonadotropin 48 h later (hCG, Alcyon, France). Superovulated females were mated with adult males (1 male/2 females) and euthanatized at 0.5 d *post coitum* (usually between 10 and 11 am). Oviduct were dissected, and the ampulla nicked to release zygotes associated with surrounding cumulus cells into a 200 µL droplet of hyaluronidase (Sigma) in M2 solution (300 µg/ml, Sigma) under a stereomicroscope (Olympus SZX9). Zygotes were incubated for 1 min at room temperature and passed with a mouth pipette through 3 washes of M2 medium to remove cumulus cells. Zygotes were kept in M16 medium (Sigma) in a water jacketed CO_2_ incubator (5% CO_2_, 37 °C) until electroporation, which was usually performed between 2 to 3 pm.

### Guide RNA design

Design was optimized to maximize cutting specificity and efficiency, using the online CRISPOR freeware (http://crispor.tefor.net/)^31^. We selected sequences (20 nt) which were unique in the genome, avoiding the presence of predicted off-target sites on the same chromosome as on-targets. Except for one case discussed below, all predicted off-targets were unlikely as they displayed at least 3 (one case) or 4 mismatches with on-target sequences, partly localized close to the PAM, which is more detrimental for off-target cleavage (Supplementary Table S2). The exception was for the achievement of MCT8 amino-acid substitution that left no latitude in choosing the guide RNA^32^. A predicted site for off-target mutation was located in an intronic sequence on the same X-chromosome as on-target, with 3 mismatches all residing distally to PAM (non-seed region). The likelihood of this unintended target to be cut was thus increased. Although this off-target site was localized 60 Mb away from on-target and thus could segregate during mice mating, we verified by PCR/Sanger sequencing that no off-target mutation was transmitted at this position (Supplementary Fig. S4).

### Preparation of RNP components and ssODN

Recombinant SpCas9 (PNABio, reference CP02) was dissolved in 1 mM DTT (5 µg/µL; 30 µM). 2 µL aliquots were stored at −80 °C for up to one year. Custom RNA synthesis was ordered either from TriLink (tracrRNA; PAGE and HPLC purification) or from Eurogentec (crRNA; HPLC purification). 2′-O-methyl +3′phosphorothioate chemical modifications were introduced on nt 1 to 3 of crRNA and nt 69 to 71 of tracrRNA. All sequences are on Supplementary Table S1. crRNA and tracrRNA were dissolved in ultrapure water (2 µg/µL). 15 µL of a 1 µg/µL equimolar mix of tracrRNA+ crRNA (4.8 µL of tracrRNA + 2.7 µL of crRNA + 7.5 µL H_2_O) were prepared and filtrated on a Millex-GV 0.22 µm filter (Millipore). The absence of contaminating RNase in these dual guide RNA preparations was ascertained as follows: 2 µL of annealed RNA were diluted in H_2_O (1/10). Half of this sample was incubated at 37 °C for 1 h to favor possible contaminating RNase activity. The migration on a 2% agarose gel of this sample was then compared to the migration of the non-incubated control. Clear bands with equivalent intensity and without smearing should be visible for both samples. Dual-guide RNA preparations which passed the quality control test were aliquoted (4 µL) and stored at −80 °C. The design of the ssODN used as template for HDR was performed following published recommendations^33^: strand choice was such that ssODN sequence was complementary to crRNA 5′ end (copying the “non PAM strand”), and the position of the ssODN sequence was asymmetric with respect to the double strand break, displaced toward the 3′ region. Size of 5′ homology was 92 nt and 36 nt for 3′ homology. Phosphorothioate modifications were introduced at positions 1, 2, 3, 136, 137 and 138 to protect the ssODN from nucleases^34^. ssODN purified by PAGE (Eurogentec) was resuspended in ultrapure water (4 µg/µL) and filtrated on a Millex-GV 0.22 µm filter.

### RNPs assembly

RNP assembly was performed just before electroporation. One aliquot of 4 µg RNA mix (4 µL) was incubated at 80 °C for 2 min for denaturation, and then at 37 °C for 10 min to favor annealing of the tracrRNA and crRNA into a dual guide RNA. 10 µg of Cas9 protein (2 µL) were added before additional 10 min incubation at room temperature for RNP complex formation. When two RNPs were electroporated, the amount of each dual-guide RNA was reduced by half. When needed, 4 µg (1 µL) of ssODN was finally included. The final volume was adjusted to 20 µL with Opti-MEM (Life Technologies). This provides enough RNPs solution for the electroporation of at least 3 batches of 20–30 zygotes each.

### Electroporation of pronuclear-stage embryos with intact zona pellucida

We adapted a procedure consisting in repeated pulses of electroporation delivered by a NEPA 21 electroporator (NEPA GENE Co. Ltd., Chiba, Japan) to first damage the *zona pellucida* and then favor the intracellular entry of RNPs^18^. A glass chamber with 1 mm gap platinum plate electrodes (CUY5001P1–1.5, NEPA GENE Co. Ltd) was filled with 5 μL of RNPs-containing medium, with or without ssODN. Between 15 and 30 zygotes were aligned in the chamber, taking care to avoid contacts between zygotes and electrodes. Impedance was measured and maintained between 120 and 180 kΩ by liquid volume adjustment (reducing volume increases impedance). Four “poring pulses” were applied (40 V, 3.5 ms, interval 50 ms, 10% voltage decay, polarity+), followed by 5 “transfer pulses” (5 V, 50 ms, interval 50 ms, 40% voltage decay; alternating + and − polarity). Poring pulses worked equally well when voltage was between 30 V and 50 V (in the latter case, duration was reduced to 2.5 ms). Zygotes were then transferred into M16 medium and kept overnight in incubator. The embryos that reached the 2-cell stage (89 to 97%, Table 2) were transferred into the oviduct of B6CBAF1 (Charles River, France) pseudopregnant females (15–20 embryos per female). Embryo mortality, comparing the number of newborns to the number of transferred embryos, was similar to the one usually observed after microinjected embryo transfer, and was not significantly affected by the electroporation procedure (Table 2).

### Genotyping

PCR amplifications were performed directly on alkaline lysates of toe clips. Briefly, each toe was incubated 30 min at 95 °C in 100 µL of alkaline solution (NaOH 25 mM, EDTA 0.2 mM, pH 12.0). Neutralization was performed by adding 100 µl Tris 40 mM, pH 5.0. Two microliters of lysates were analyzed by PCR (25 µL) with appropriate primers. Large deletions were identified by agarose gel electrophoresis. Small indels and point mutations were identified by Sanger sequencing of PCR products (Genewiz). For indels’ identification, Sanger electrophoretograms obtained from mosaic F0 mice were analyzed using the TIDE software^35^ which decomposes sequencing traces and estimates the respective contribution of each PCR fragment. Indel size that can be identified by TIDE is limited to 50 nt, therefore this software was not used for mice with bigger indels as detected by agarose gel electrophoresis. TIDE analysis becomes difficult when spacing between the nucleotides on the Sanger electrophoretogram is not constant. In this case, TIDE appends a “spacing not constant” message to the result.

### Data availability

All data generated or analyzed during this study are included in this published article and its Supplementary Information file.

## Electronic supplementary material

Supplementary Information
