# Author Correction: Electroporation of mice zygotes with dual guide RNA/Cas9 complexes for simple and efficient cloning-free genome editing

**DOI:** 10.1038/s41598-018-22724-9

**Published:** 2018-03-13

**Authors:** Marie Teixeira, Bénédicte F. Py, Christophe Bosc, Daphné Laubreton, Marie-Jo Moutin, Jacqueline Marvel, Frédéric Flamant, Suzy Markossian

**Affiliations:** 1 SFR BioSciences, Plateau de Biologie Expérimentale de la Souris (AniRA-PBES), Ecole Normale Supérieure de Lyon, Université Lyon1, CNRS UMS3444, INSERM US8, 69007 Lyon, France; 2 CIRI, INSERM U1111, Université Claude Bernard Lyon 1, CNRS UMR 5308, École Normale Supérieure de Lyon, Université de Lyon, 69007 Lyon, France; 3 Université Grenoble Alpes, Grenoble Institut des Neurosciences, GIN, F-38000 Grenoble France; 4 INSERM, U1216, F-38000 Grenoble, France; 5 Institut de Génomique Fonctionnelle de Lyon, INRA USC 1370, Université de Lyon, Université Lyon 1, CNRS UMR 5242, Ecole Normale Supérieure de Lyon, 46, allée d’Italie, 69007 Lyon France

Correction to: *Scientific Reports* 10.1038/s41598-017-18826-5, published online 11 January 2018

This Article contains typographical errors.

In the Methods section,

“Impedance was measured and maintained between 120 and 180 kΩ by liquid volume adjustment (reducing volume increases impedance)”

should read:

“Impedance was measured and maintained between 120 and 180 Ω by liquid volume adjustment (reducing volume increases impedance)”.

In Table 1,

“Adjust volume to keep impedance between 120 and 180 kΩ”

should read:

“Adjust volume to keep impedance between 120 and 180 Ω”.

