# Supplementary material for: Electroporation of mice zygotes with dual guide RNA/Cas9 complexes for simple and efficient cloning-free genome editing
Source: Sci Rep. 2018 Jan 11;8:474. doi: 10.1038/s41598-017-18826-5 (PMC5764989; doi:10.1038/s41598-017-18826-5)
Supplement: Supplementary file 1 — Supplementary Information [file 41598_2017_18826_MOESM1_ESM.pdf]

Supplementary Information

Electroporation of mice zygotes with dual guide RNA/Cas9 complexes for simple and efficient cloning-free genome editing.

Marie Teixeira[1], Bénédicte F Py[2], Christophe Bosc[3,4], Daphné Laubreton[2], Marie-Jo Moutin[3,4,], Jacqueline Marvel[2], Frédéric Flamant[5], Suzy Markossian[5*]

1 SFR BioSciences, Plateau de Biologie Expérimentale de la Souris (AniRA-PBES), Ecole Normale Supérieure de Lyon, Université Lyon1, CNRS UMS3444, INSERM US8, France

2 CIRI, INSERM U1111, Université Claude Bernard Lyon 1, CNRS UMR 5308, École Normale Supérieure de Lyon, Université de Lyon, 69007 Lyon, France.

3 Université Grenoble Alpes, Grenoble Institut des Neurosciences, GIN, F-38000 Grenoble, France

4 Inserm, U1216, F-38000 Grenoble, France

5 Institut de Génomique Fonctionnelle de Lyon, INRA USC 1370, Université de Lyon, Université Lyon 1, CNRS UMR 5242, Ecole Normale Supérieure de Lyon, 46, allée d'Italie 69364 Lyon cedex 07 France

*To whom correspondence should be addressed. Tel: 00 (33) 4 26 73 13 31 Email: Suzy.Markossian@ens-lyon.fr

Supplementary Figure S1

a)

```
GTTGGACGAGCCGCTGTGCGCCCTTGGCGGACGTGAGCGGAAAGAAGATGGCGGTGCAGGTGGTGCAAGCTGTGC
AGGCGGTTCATCTTGAGTCTGACGCTTTCCTAGTTTGTCTCAACCATGCTCTGAGCACAGAAAAGGAGGAAGTGA
TGGGTCTGTGTATAGGGGAGGTAAGTTGGCCACCTTGGCTGAAATCTTGCTGATCAATACCCTGTGTTCCTGGAG
GCGGCTGGGAGGCCATAGGGATGTGATTTGGATCCTTTGAGGTGTTTTATTTTGTTGTTCGAGACAGGGTTTCTC
TGTGTAGCCCCGGCTGTCCTGGAACTCACGCTGTAGACCAGGCTGGCCTCGAACTCAGAAATCCGCCTGCCTCTG
CCTCCCGAGTGCTGGGATTCCGGCTTCCTTTGAGTTTTTAATGCTGTCGCGGGGTACTTTTCTTCTGGCGCTCTG
ATCCATGCTTATGTTCCTGTAAACCCTCAGCGGTCTGCATGAACTCAGGGGATGGGGGTTATCATACCAGTGATT
AAAACTGCAAACCAGGCCAGAAACACATTCTGGCATGAGTTATCCAGATATTAGAAAACCAGTAGCCCAGGTTAC
```

b)

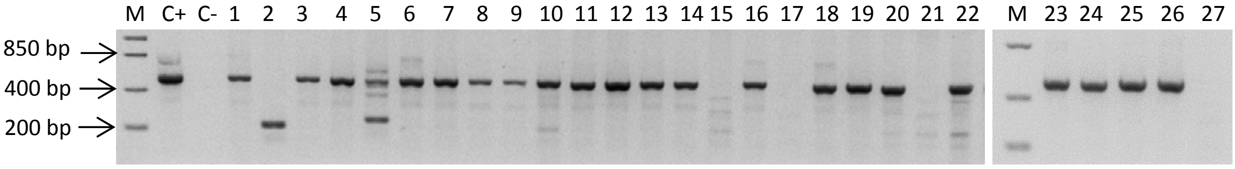

c)

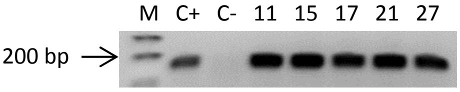

d)

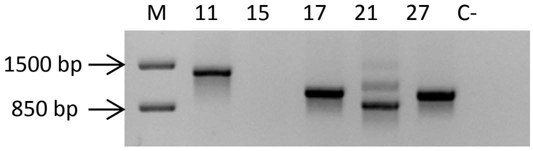

Supplementary Figure S1. ***Brcc3 gene knock-out*** **experiment. a)** Targeted gene sequence: sequence targeted by the crRNA 5'end is underlined; PAM (3nt) and PCR primers are shaded. **b)** PCR analysis of edited *Brcc3* gene by 2% agarose gel electrophoresis. Samples 1 to 27: PCR performed on F0 mice genomic DNA. Samples 15, 17, 21 and 27 repeatedly failed to amplify. **c)** The integrity of DNA samples 15, 17, 21 and 27 was verified by amplifying 130 bp of another locus (*Nlrp3*). Sample 11 was used as a positive control. Confirmed integrity of these samples suggests the occurrence of large deletions at the targeted locus. **d)** Larger deletions were searched by using primers more distal to the cut site and confirmed in samples 17, 21 and 27. Control sample n°11 showed the expected 1358 bp fragment. M: molecular weight marker (DNA Ladder Fast, Low Range, Euromedex). Sample C+ is a control PCR of C57BL/6 genomic DNA. Sample C– is a negative control without genomic DNA.

Supplementary Figure S2

a)

Vash1

CCCCACCTCCTGATCATCGTCCCACCCCCGTTGAGGATTTAGGGATGCCAGGGGGAAAGAAGGTGGTCCCAAGTG
GCAGCAGCAGTGCCTCCCCGAACGCAGCCGCCACCACCACTGCTGCTGCTGCTGCTGCCGCTGCTGCCCCCCACT
CTGGCACTAAACGTTTGGAGACCACCGAAGGGGCCTCAGCACAGAGAGATGAGGAACCCGAAGAGGAAGGGGAAG
AGGACCTACGAGATGGAGGTGTCCCTTTTTTCATCAACCGAGGTGGACTGCCAGTGGATGAGGCCACCTGGGAAA
GGATGTGGAAGCATGTGGCCAAGATCCACCCAGATGGAGAGAAGGTGGCCCTGCGCATCCGTGGGGCCACCGACC
TGCCCAAGGTAAGACATGGAGGGGCCAGGCACAGTGAAGATGGGTTTTATTGACCTTGAAGCTAAGAAAGTAACC

Vash2

CTTAGCCAGGAAGGCAGCGTTTCTGATGGCGCCTACCATCTCTCCCCCTCTGGGGTGGACCTTGGCAACGTGCAG
CCACATCCGCTCCCAGGTGTGGCTATCGATCGGGAACCCGCTCTTGTTCACGTGGAACAGCACCCCGCCGTCTTT
GTCCTCCTCCTCGGAGCCCCCGCTGGTGGCGAGGCTCACGGGCCGCGCGTGGCTGCTCCGGGATCGGGTGCCTTT
GGTGATTTTGGGGTGGGGGCAGCGGTGAGTGTCGGCGGCAGAGCCGGTCATGATGAGGGGCACACGGCAAGGGGG
CGGAGGACGCCGGCACGCCGGGGATCAGGGCCCCGCGGGAGGTTGGGGTGGCCTTTAACTCTCGCGGCCGTAGGA
CACACGAAGGGAACCCCGCTCACCGACCGGGCCTGAAAGCAGTGGAAACAGCAGACACTGCAGCTCAATAAGACA

b)

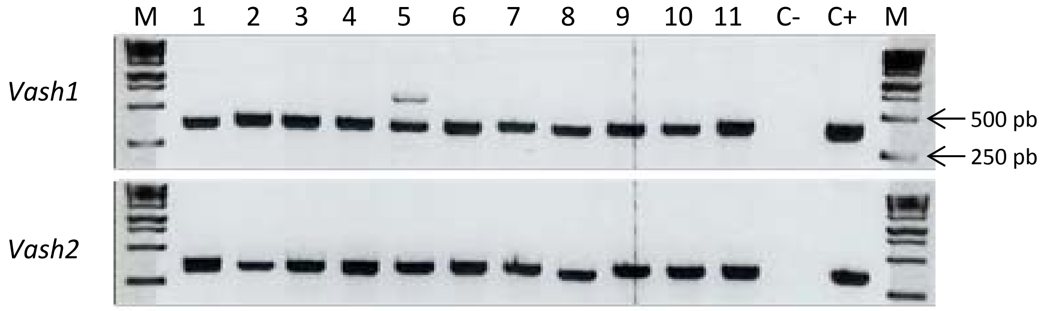

Supplementary Figure S2. *Vash1 & Vash2* gene editing experiment. **a)** *Vash1 and Vash2* targeted sequences: sequences targeted by the crRNA 5’end are underlined, PAM (3 nt) and PCR primers are shaded. **b)** PCR analysis of edited *Vash1* and *Vash2* genes by 2% agarose gel electrophoresis. Samples 1 to 11: PCR performed on F0 mice genomic DNA. For *Vash1*, note the additional band for mice n°5. M: molecular weight marker (1kb DNA Ladder ready-to-use, Euromedex). Sample C+ is a control PCR of C57BL/6 genomic DNA. Sample C– is a negative control without genomic DNA.

Supplementary Figure S3

a)

AAAGCCATTGCTGCTTCTGGTCTTGAAGGTTCCCTCCTGGGTCTTGCTGTAGTCAGGCTCTGAACAGGTATAGAG
GCAATGGGAGCCTTTATTTATAAATGCCCCTTTATTTCCTGATCTTTCTCCAGAGTGCCCCTCAGAAGACATCAG
TCCCTTCG*GAAGAAACTACGGGCCCAAGGACAGCTCTCAGAATTCTGGAGGTCTCATAACTTGGACATGACCCGA*
*CTCAGCGAGTCCTGTA*ATGTGTATTCGAGTGTCAATGAACCCCTCATCAACTACCTGGATGTAAGATTTCTCAAA
TTGAAGCCTGAGGACACTGGGTGTAGGGAGGAAGGCTGGGGCCTTCAGCAGGGGCCAGTGCTGCATTGGTGGCAT
TATGATCAGGCATGGATTTGTTTGCTTGGAGGATTCAGACAGTCCCCCAGTCTCTATCTACTCTGTAAAGCAGGC
TTCTAAATGACCCAGTATGCAGAGCTTTCTTCAAGAGGAAAGAGATAAGTATGATATGTGTTCCAAAAAGAAAGA

b)

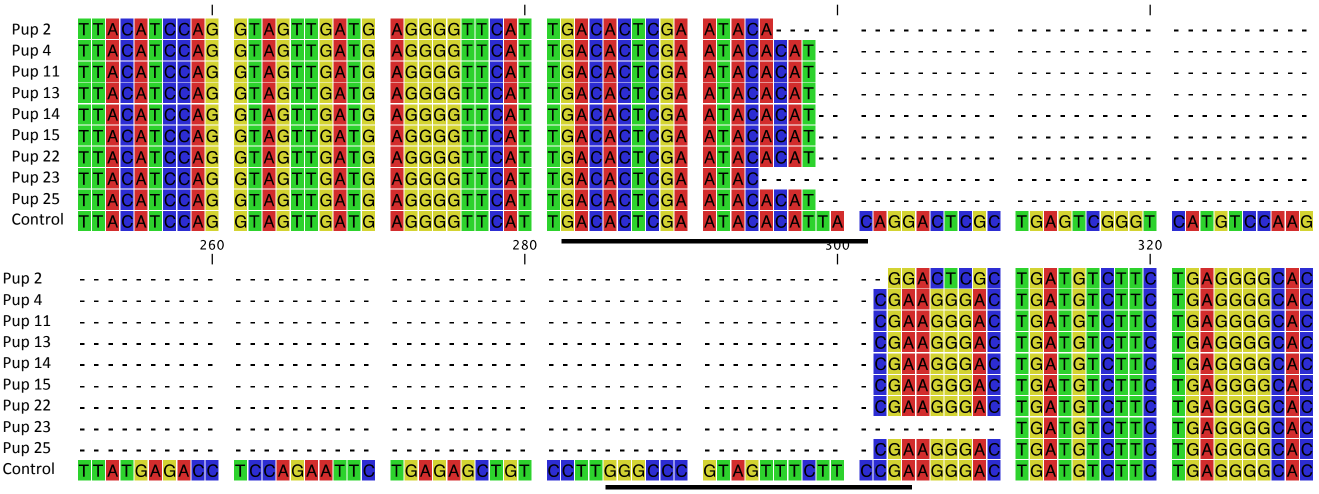

c)

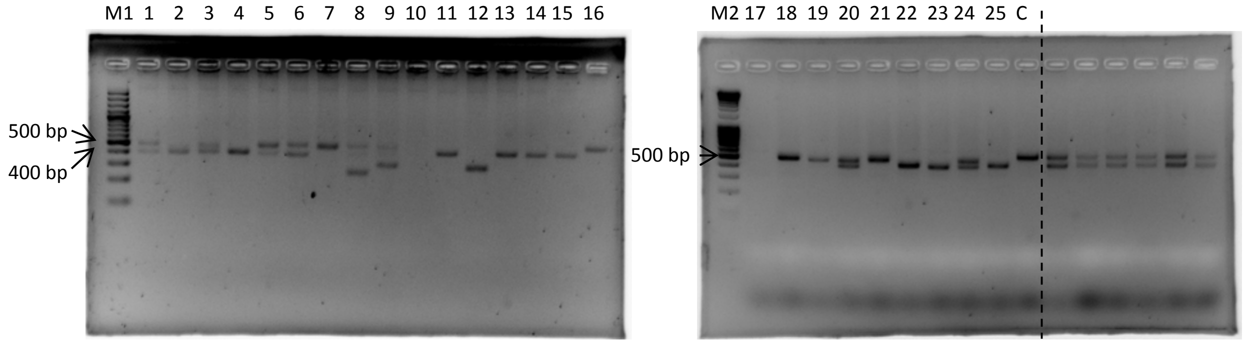

d)

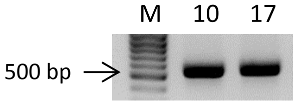

Supplementary Figure S3. *Cste* gene 83 pb deletion. **a)** Targeted sequence: deletion (italic) is expected between the two sequences targeted by two crRNAs (underlined). PAM (3 nt) and PCR primers are shaded. **b)** Alignment of sequencing results (reverse complement) from control B6.SJL-PtprcaPepcb/BoyCrl mouse and F0 mice n°2-4-11-13-14-15-22-23-25. Underlined nucleotides on control sequence correspond to guide RNA sequences. In these 9 mice, Sanger sequencing of PCR products identifies a single mutant allele, corresponding exactly to the expected deletion, some with few nucleotides difference (n°2 and 23). **c)** Full length gels of cropped gels presented in Fig2. A fragment of 481 bp is expected for wild-type alleles, whereas 398 bp fragment is expected for deleted alleles. The six last lanes of the second gel separated by a dashed lane are from another experiment. **d)** PCR confirming DNA integrity for samples 10 and 17 that failed to amplify with *Cste* primer: an independent locus was successfully amplified using Mct8-For and Mct8-Rev primers. This suggests the occurrence of larger deletions at the targeted locus. M1 and M2: molecular weight marker (respectively O'GeneRuler 100bp Plus DNA Ladder and MassRuler DNA Ladder Mix, ThermoFisher Scientific).

Supplementary Figure S4

a)

TCTGCTCGGCAGGTTGGTGGCGAGTGTGGCTTTGGGTAAAGGTGGAGGAATGGAGGTTTCCAAGAACA
AAGGGGGCAGGTAAGTTGCTTTCAGGCCGCTGAAGATAGCTCTTGGTGTTTCTCCCACAGCTCCCTAA
GCCTGCGCTACTTCACCTATGGGATTCTGTTTGGTTGTGGCTGCTCATTCGCCTTTCAACCATC**ACTC**
GTCATCCTGGGCCACTACTTTCAACGTCGCCTAGGTCTAGCCAATGGTGTGGTATCTGCTGGAAGTAG
CATCTTCTCCATGTCTTTTCccTTccTcATcAAAATGCTGGGGGATAAAATCAAGCTGGCCCAAACCT
TCCAGGTGCTGAGTACCTTCATGTTTGTCCTTACGCTGCTCTCACTCACTTACCGGCCCCTCCTGCCA
AGCTCCCAGGACACCCCAAGCAAGAGAGGTGCCCACACCCTGCGACAGCGCTTTCTGGTTCAGTTCAG
AAAGTACTTCAACATGCGTGTATTCCGCCAGCGCACTTACCGTATCTGGGCCTTTGGGATCGCTGCTG
CTGCCCTTGGTTACTTCGTCCCCTACGT

b)

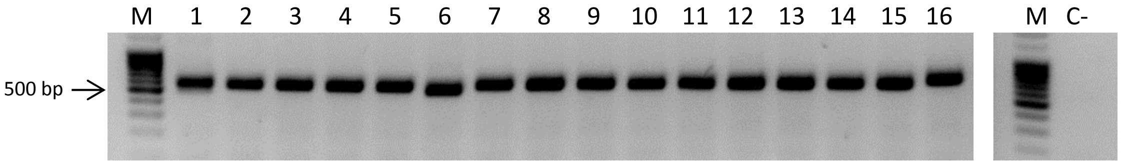

c)

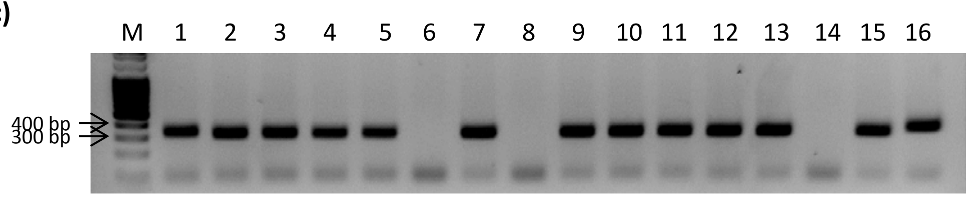

d)

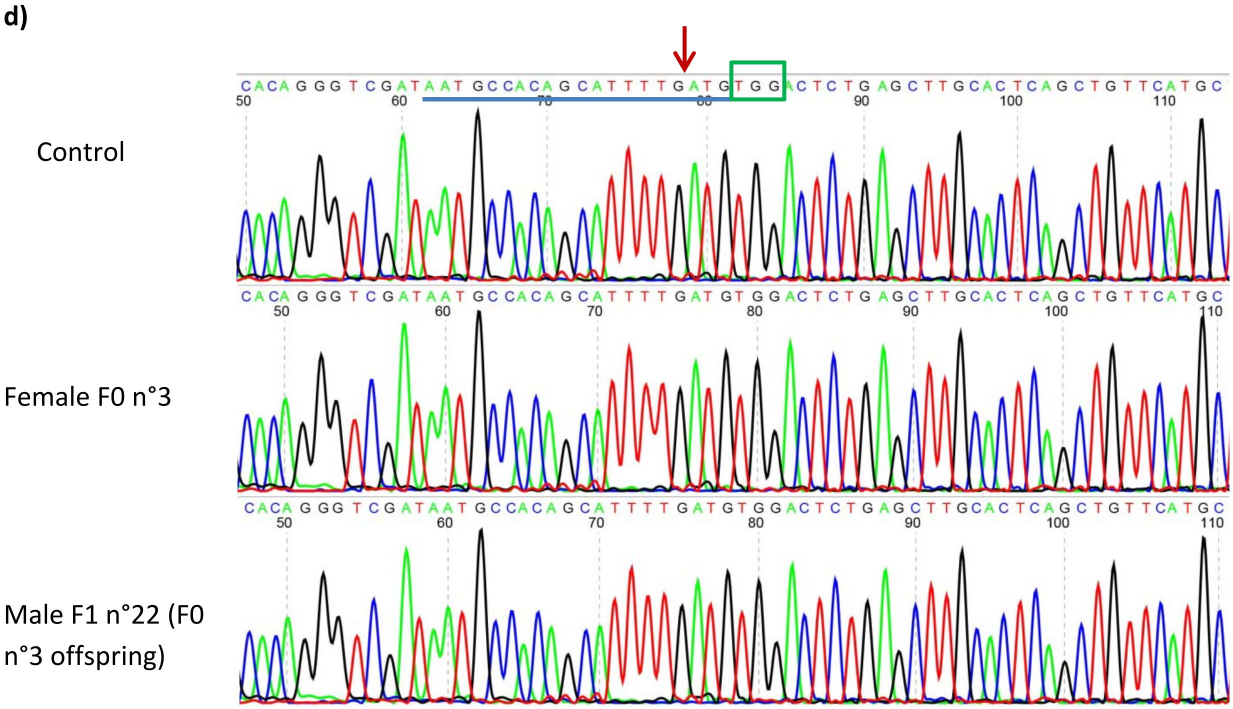

Supplementary Figure S4. Amino-acid substitution in the MCT8 transporter using HDR. a) *Mct8* gene targeted sequence: sequence targeted by the crRNA is underlined. PAM (3 nt) and PCR primers are shaded. Sequence covered by the ssODN is bolded and nucleotides which are mutated through HDR with ssODN are in lower case letters. **b)** PCR analysis of F0 mice genomic DNA on 2% agarose gel: PCR used for Sanger sequencing (Mct8-For & Rev primers, 537 pb). Sanger sequencing analysis showed mutations for all pups (NHEJ or HDR). M: molecular weight marker (MassRuler DNA Ladder Mix, ThermoFisher Scientific). C-: negative control with no DNA. **c)** PCR of F0 mice genomic DNA with mutation-specific primers (Mct8-For & Mct8PM-Rev primers, 309 pb) analyzed on 2% agarose gel. All mice except n°6-8 and 14 seem to have integrated point mutations. Subsequent sequence analysis revealed that n°1 and 16 were repaired by partial integration of ssODN at the cut site. Of note, no other allele was detected for 8 pups out of 11 that underwent correct HDR repair. M: molecular weight marker (MassRuler DNA Ladder Mix, ThermoFisher Scientific). **d)** Control of a potential intronic off-target site in *Prps2* gene: DNA Sanger sequencing of control FVBxB6D2F1 mouse, mutant F0 female n°3 and one of its mutant male offspring (n°22) are shown. Top chromatogram represents a part of wild-type *Prps2* gene: potential off-target site is underlined in blue, green rectangle indicates PAM, red arrow indicates Cas9 potential off-target cutting site. Bottom chromatograms represent mutant mice F0 n°3 and F1 n°22, in which no mutation were detected.

Supplementary Figure S5

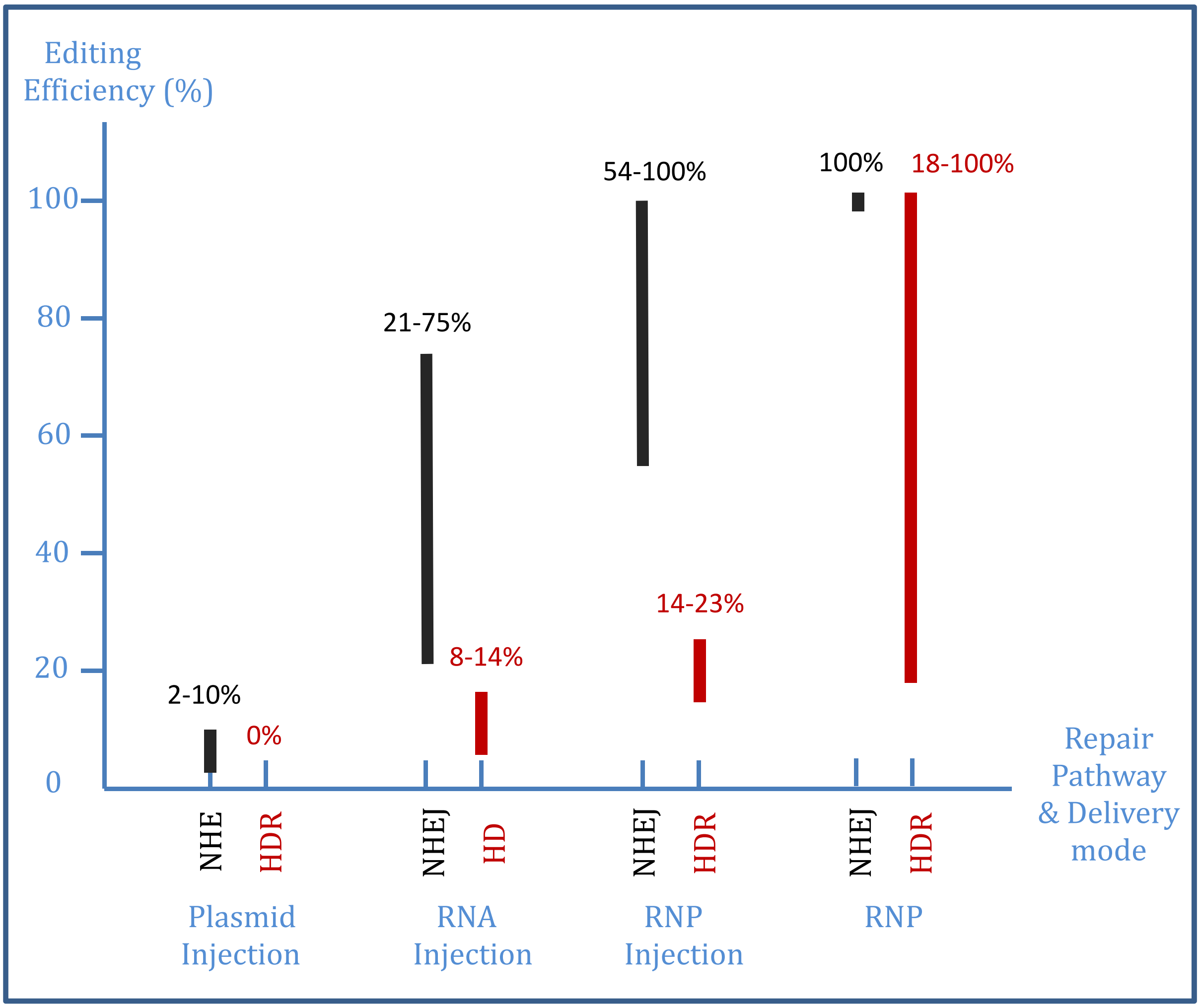

Supplementary Figure S5. Preliminary comparison between different CRISPR/Cas9 genome editing protocols. Efficiencies obtained with different delivery mode of CRISPR/Cas9 components in our facility over the last years (% of mutated animals/newborn). For plasmid injections (pX330 plasmid from Addgene +/- ssODN): 1 experiment used NEHJ repair mechanism (10% efficiency), 1 experiment used HDR (no HDR and 2% NHEJ achieved). For RNA injections (Cas9 mRNA (TriLink) and *in vitro* transcribed sgRNA +/- ssODN): 2 experiments used NEHJ repair mechanism, 3 experiments used HDR. For RNP injections (Cas9 protein complexed to synthetic tracrRNA/crRNA chemically modified +/- ssODN): 6 experiments used NEHJ repair mechanism, 2 experiments used HDR. For RNP electroporation (Cas9 protein complexed to tracrRNA/crRNA +/- ssODN as described in the present study): 4 experiments used NEHJ repair mechanism, 4 experiments used HDR (18, 69, 86 and 100% HDR efficiency). Asymetric ssODN donors were used in the last three HDR experiments.

Supplementary Table S1. Oligonucleotides used in the study

	Name	Sequence	PCR size (bp)
	tracrRNA	5’AAACAGCAUAGCAAGUUAAAAUAAGGCUAGUCCGUUAUCAACUUGAAAAAG UGGCACCGAGUCGGUGC[U*U*U*]U3'	
Brcc3	Brcc3-crRNA	5'[A*G*U*]GAUGGGUCUGUGUAUAGGUUUUAGAGCUAUGCUGUUUUG3'	
	Brcc3-Forward	GTGAGCGGAAAGAAGATGGC	514
	Brcc3-Reverse	TCTGGCCTGGTTTGCAGTTT	
	Brcc3-Seq For	GTGAGCGGAAAGAAGATGGC	
	Brcc3_9_F	CGAGCCGAGGTCACTAGAAC	1358
	Brcc3_11_R	GGCCATATGTTCACACACAACA	
	Nlrp3-F	ATCCTAGGTTTCTCTGAGGCCAA	130
	Nlrp3-R	GGGGGATGAAGCACATGGTAA	
Vash1 & *Vash2*	Vash1-crRNA	5’[C*C*A*]CTCTGGCACTAAACGTTGUUUUAGAGCUAUGCUGUUUUG3’	
	Vash2-crRNA	5’[U*C*A*]CCAAAGGCACCCGAUCCGUUUUAGAGCUAUGCUGUUUUG3’	
	Vash1-Forward	CTCCTGATCATCGTCCCACC	394
	Vash1-Reverse	GCCCCTCCATGTCTTACCTT	
	Vash1-SeqFor	TTGAGGATTTAGGGATGC	
	Vash2-Forward	CGTTTCTGATGGCGCCTAC	373
	Vash2-Reverse	GGTTCCCTTCGTGTGTCCTA	
	Vash2-SeqFor	GCGCCTACCATCTCTCCC	
Ctse deletion	Ctse-crRNA-1	5'[G*G*G*]CCCGUAGUUUCUUCCGAGUUUUAGAGCUAUGCUGUUUUG3'	
	Ctse-crRNA-2	5'[G*A*C*]ACUCGAAUACACAUUACGUUUUAGAGCUAUGCUGUUUUG3'	
	Ctse-Forward	TTGCTGCTTCTGGTCTT	481/398
	Ctse-Reverse	CCTCTTGAAGAAAGCTCT	
	Ctse-Seq Rev	CCTCTTGAAGAAAGCTCT	
Mct8 point mutations (note1)	Mct8-crRNA	5'[U*A*U*]CCCCCAGCAUUUUGAUGGUUUUAGAGCUAUGCUGUUUUG3'	
	Mct8-ssODN	5'A*C*T*CGTCATCCTGGGCCACTACTTTCAACGTCGCCTAGGTCTAGCCAA TGGTGTGGTATCTGCTGGAAGTAGCATCTTCTCCATGTCTTTTCtgTTtttTaA TtAAAATGCTGGGGGATAAAATCAAGCTGGCCCAA*C*C*C3'	
	Mct8-For	AGGTTGGTGGCGAGTGTGG	537
	Mct8-Rev	CAGCAGCAGCGATCCCAAAG	

	Mct8-SeqFor	AGGTTGGTGGCGAGTGTGG	
	Mct8PM-Rev	TATCCCCCAGCATTTTaATtAaaAAc	309
	Prps2 OT seq	AATGCCACAGCATTTTGATG(TGG)	
	Prps2OT-F2	ACAGTGGATTGCGAGCCAGG	884
	Prps2OT-R2	CTGCTCCAGGTCCTCAGATATGG	
	Prps2OT-seqF	GACTTGATGAGATTTCTCACTG	

Note 1: lower case letters in ssODN and PCR primer are for mutated nt. * are for phosphothiorate and [ ] for 2'-O-methyl chemical modifications. OT: off-target; mismatches compared to on-target are underlined; ( ) PAM sequence.

Supplementary Table S2

guide RNA	Number of predicted exonic OT	Number of predicted intronic or intergenic OT (number of MM)	Number of predicted OT with higher likelihood (MM only located outside seed sequence)
Brcc3-crRNA	0	12 (4 MM)	0
Vash1-crRNA	0	4 (4 MM)	0
Vash2-crRNA	0	0	0
Ctse-crRNA-1	0	1 (4 MM)	0
Ctse-crRNA-2	0	1 (3 MM)	0
		6 (4 MM)	0
Mct8-crRNA	0	**1 (3 MM)**	**1**
		4 (4 MM)	0

Supplementary Table S2. Analysis of potential off-target sites located in the same chromosome as on-targets. First, no potential off-target sequence was predicted within exons. Second, most of intronic or intergenic predicted off-target sites were unlikely as they had 3 or 4 mismatches compared to on-targets, with one or more of these mismatches residing within seed sequence (PAM proximal). Finally, one potential off-target site with 3 mismatches all located outside seed sequence (PAM distal) was predicted (bold). We thus assayed for the occurrence of this more likely off-target mutation (Supplemental Figure S4). OT: off-target; MM: mismatch.
